# Associations Between Measures of Balance and Lower-Extremity Muscle Strength/Power in Healthy Individuals Across the Lifespan: A Systematic Review and Meta-Analysis

**DOI:** 10.1007/s40279-015-0390-z

**Published:** 2015-09-28

**Authors:** Thomas Muehlbauer, Albert Gollhofer, Urs Granacher

**Affiliations:** Division of Training and Movement Sciences, Research Focus Cognition Sciences, University of Potsdam, Am Neuen Palais 10, Building 12, 14469 Potsdam, Germany; Albert-Ludwigs-University Freiburg, Institute of Sport and Sport Science, Freiburg, Germany

## Abstract

**Background:**

It has frequently been reported that balance and lower-extremity muscle strength/power are associated with sports-related and everyday activities. Knowledge about the relationship between balance, strength, and power are important for the identification of at-risk individuals because deficits in these neuromuscular components are associated with an increased risk of sustaining injuries and falls. In addition, this knowledge is of high relevance for the development of specifically tailored health and skill-related exercise programs.

**Objectives:**

The objectives of this systematic literature review and meta-analysis were to characterize and, if possible, quantify associations between variables of balance and lower-extremity muscle strength/power in healthy individuals across the lifespan.

**Data Sources:**

A computerized systematic literature search was performed in the electronic databases PubMed, Web of Science, and SPORTDiscus up to March 2015 to capture all relevant articles.

**Study Eligibility Criteria:**

A systematic approach was used to evaluate the 996 articles identified for initial review. Studies were included only if they investigated healthy individuals aged ≥6 years and tested at least one measure of static steady-state balance (e.g., center of pressure [CoP] displacement during one-legged stance), dynamic steady-state balance (e.g., gait speed), proactive balance (e.g., distance in the functional-reach-test), or reactive balance (e.g., CoP displacement during perturbed one-legged stance), and one measure of maximal strength (e.g., maximum voluntary contraction), explosive force (e.g., rate of force development), or muscle power (e.g., jump height). In total, 37 studies met the inclusionary criteria for review.

**Study Appraisal and Synthesis Methods:**

The included studies were coded for the following criteria: age (i.e., children: 6–12 years, adolescents: 13–18 years, young adults: 19–44 years, middle-aged adults: 45–64 years, old adults: ≥65 years), sex (i.e., female, male), and test modality/outcome (i.e., test for the assessment of balance, strength, and power). Studies with athletes, patients, and/or people with diseases were excluded. Pearson’s correlation coefficients were extracted, transformed (i.e., Fisher’s *z*-transformed *r*_*z*_ value), aggregated (i.e., weighted mean *r*_*z*_ value), back-transformed to *r* values, classified according to their magnitude (i.e., small: *r* ≤ 0.69, medium: *r* ≤ 0.89, large: *r* ≥ 0.90), and, if possible, statistically compared. Heterogeneity between studies was assessed using *I*^2^ and Chi-squared (*χ*^2^) statistics.

**Results:**

Three studies examined associations between balance and lower-extremity muscle strength/power in children, one study in adolescents, nine studies in young adults, three studies in middle-aged adults, and 23 studies in old adults. Overall, small-sized associations were found between variables of balance and lower-extremity muscle strength/power, irrespective of the age group considered. In addition, small-sized but significantly larger correlation coefficients were found between measures of dynamic steady-state balance and maximal strength in children (*r* = 0.57) compared with young (*r* = 0.09, *z* = 3.30, *p* = 0.001) and old adults (*r* = 0.35, *z* = 2.94, *p* = 0.002) as well as in old compared with young adults (*z* = 1.95, *p* = 0.03).

**Limitations:**

Even though the reported results provided further insight into the associations between measures of balance and lower-extremity muscle strength/power, they did not allow us to deduce cause and effect relations. Further, the investigated associations could be biased by other variables such as joint flexibility, muscle mass, and/or auditory/visual acuity.

**Conclusions:**

Our systematic review and meta-analysis showed predominately small-sized correlations between measures of balance and lower-extremity muscle strength/power in children, adolescents, and young, middle-aged, and old adults. This indicates that these neuromuscular components are independent of each other and should therefore be tested and trained complementarily across the lifespan. Significantly larger but still small-sized associations were found between measures of dynamic steady-state balance and maximal strength in children compared with young and old adults as well as in old compared with young adults. These findings imply that age/maturation may have an impact on the association of selected components of balance and lower-extremity muscle strength.

## Key Points

**Table:** 

The present systematic review and meta-analysis characterized and quantified associations between measures of balance and lower-extremity muscle strength/power in healthy individuals across the lifespan (≥6 years).
Irrespective of the investigated age group, our analyses revealed predominately small-sized correlations between measures of balance and lower-extremity muscle strength/power.
The primarily small-sized correlations between proxies of balance and lower-extremity muscle strength/power indicate that these components are independent of each other (i.e., task-specific) and should therefore be tested and trained complementarily across the lifespan.
The observed age-related differences in associations between measures of dynamic steady-state balance and maximal strength imply that maturity and biological aging may have an impact on selected components of balance and strength.

## Introduction

Balance and muscle strength/power represent important health and skill-related components of physical fitness that have to be sufficiently developed across the lifespan to successfully perform sport and everyday activities without suffering injuries and falls [1]. In contrast, deficits in balance and lower-extremity muscle strength/power have been identified as important intrinsic (person-related) injury and fall risk factors in children [2], adolescents [3], adults [4], and seniors [5]. For example, Wang et al. [3] showed that large postural sway during one-legged stance was associated with a significant increase in the risk of sustaining ankle injuries (odds ratio [OR] = 1.2) in high school basketball players aged 17 years. Furthermore, deficits in lower-extremity muscle strength (i.e., isokinetic knee flexor/extensor strength asymmetry) were identified as a significant risk factor (OR = 3.9) for non-contact quadriceps and hamstrings strains in young soccer players aged 19–28 years [4]. Another meta-analysis revealed that low levels of eccentric inversion strength (relative risk [RR] = −0.34), low level of postural stability (RR = 2.06), and a low level of inversion proprioception (RR = 0.57) represent relevant causes of ankle injuries in athletes [6]. In adults above the age of 60 years, a meta-analysis consisting of 16 prospective and retrospective studies indicated that lower-extremity muscle weakness (OR = 4.9), balance (OR = 3.2), and gait deficits (OR = 3.0) are associated with an increased fall risk [5].

Given that annual medical treatment costs of sports-/fall-related injuries are high [7, 8], knowledge about the relationship between balance and lower-extremity muscle strength/power are important from two perspectives: (a) testing and identifying at-risk individuals; and (b) developing and implementing individually tailored injury- and fall-prevention programs. Large-sized correlations between measures of balance and muscle strength/power of the lower-extremities imply that these neuromuscular components are interlinked and not independent of each other. Thus, performance achieved in one component (e.g., balance) can be (partly) transferred to that of the other component (e.g., lower-extremity muscle strength). In addition, training-induced gains in lower extremity muscle strength (e.g., maximal strength of plantarflexors) may have an impact on balance performance (e.g., postural sway) or vice versa. In contrast, small-sized correlations between balance and lower-extremity muscle strength/power imply that these neuromuscular components are independent of each other and may thus have to be tested and trained complementarily. There are a number of reasons that imply large-sized correlations between balance and muscle strength/power of the lower-extremities. First, it has previously been shown that deficits in balance and strength are significantly associated with the occurrence of injuries and falls [2–5] and therefore represent two important intrinsic risk factors. Second, similar neurophysiological structures appear to be responsible for the control of balance and lower-extremity muscle strength/power. For instance, information from Ia afferents is important for both the regulation of balance as well as for explosive force production through the mediation of presynaptic inhibition acting on the motor neuron [9, 10]. In addition, cortical excitability is an important mechanism responsible for voluntary muscle activations but also for the control of long latency reflexes during the performance of postural tasks [11, 12]. Third, numerous studies [10, 13, 14] proved a transfer of training-related gains from one component to the other and vice versa. For example, Gruber et al. [10] scrutinized changes in balance and strength performance following 4 weeks of balance or resistance training in healthy young adults. The authors observed significant improvements in rate of force development (RFD) after balance training and in postural sway after ballistic strength training.

In addition, age appears to be an important factor that may have an impact on associations between balance and lower-extremity muscle strength/power. Recently, it has been reported that these neuromuscular components behave in a U-shaped (i.e., postural sway) or inverted U-shaped (i.e., gait speed, maximal strength, muscle power) mode across the lifespan depending on the respective variable that is taken into consideration [15]. These age-related behavioral changes are mirrored in the underlying neurophysiological structures responsible for the control of balance and strength/power [16]. In children, the neuromuscular system is still emerging due to maturation (e.g., central nervous system myelinization) and it has not reached its full functionality yet [17, 18]. In seniors, the neuromuscular system has lost its full functionality due to, for instance, a decline in the number of sensory and motor neurons [19–21].

Thus, the aims of this systematic literature review and meta-analysis were to characterize and quantify associations between variables of balance and lower-extremity muscle strength/power in healthy individuals across the lifespan. With reference to the relevant literature [2–5, 12, 13, 15], we expected (i) large-sized associations between balance and strength/power of the lower-extremities; and (ii) that the correlations between those components are modulated by age.

## Methods

### Literature Search

We performed a computerized systematic literature search in PubMed, Web of Science, and SPORTDiscus up to March 2015. The following Boolean search strategy was applied using the operators AND, OR, NOT: ((((postural balance [MeSH] OR posture [MeSH]) AND (muscle strength [MeSH] OR power) AND (correlation study OR association OR relationship) NOT (athletes [MeSH] OR patients OR disease)))). With respect to the PubMed database, Medical Subject Headings (MeSH) were used, as indicated before. The search was limited to the English language, human species, and full-text original articles. Further, we checked the reference lists of each included article and analyzed relevant review articles [22, 23] in an effort to identify additional suitable studies for inclusion in the database.

### Selection Criteria

To be eligible for inclusion, studies had to meet the following criteria: (a) participants of the experimental groups had to be healthy subjects; (b) participants were aged 6 years and older; and (c) at least one measure of balance and lower-extremity strength/power had to be tested in the study. Studies were excluded if: (a) they investigated athletes, patients, or people with diseases; or (b) it was not possible to extract correlation coefficients from the results section (see, for example, Gomes et al. [24]), or if authors did not reply to our inquiries sent by email. Based on the predefined inclusion and exclusion criteria, two independent reviewers (TM, UG) screened potentially relevant papers by analyzing the titles, abstracts, and full texts of the respective articles to elucidate their eligibility. If no consensus was achieved between the two reviewers, a third reviewer (AG) was contacted.

### Coding of Studies

Each study was coded for the following variables: number of participants, sex, and age. Further, we coded test modalities/outcomes in tests for the assessment of balance, muscle strength, and power. In accordance with the classification of postural control introduced by Shumway-Cook and Woollacott [25], balance performance was separated into four different categories: static steady-state (i.e., maintaining a steady position while sitting or standing), dynamic steady-state (i.e., maintaining a steady position while walking), proactive (i.e., anticipation of a predicted postural disturbance), and reactive balance (i.e., compensation of a postural disturbance). Lower-extremity muscle strength was divided in measures of maximal strength (i.e., maximal voluntary force/torque of the force-/torque-time curve [MVC]), explosive force (i.e., rate of force/torque development [RFD/RTD] as indicated by the slope of the force/torque-time curve), and power (i.e., jump distance, force, height, and power). If multiple measures were reported within one of the aforementioned categories, the most representative measure was used for analysis. With regards to static steady-state balance, center of pressure (CoP) displacement during one-legged stance was defined as the most important parameter. In terms of dynamic steady-state balance, gait speed was used. Concerning proactive and reactive balance, maximal reach distance in the functional-reach-test and CoP displacements during perturbed one-legged stance were defined as the most representative outcome. In terms of muscle strength, MVC was defined as the most important variable representing maximal strength. With regards to explosive force, RFD was used, and for muscle power, countermovement jump (CMJ) height was applied. If test parameters/outcomes and/or results of correlative analyses were not reported, the authors were contacted and missing information was requested. In two cases [26, 27], authors responded and provided the respective data. If authors did not respond, the study was excluded [28].

### Statistical Analyses

Associations between variables of balance and lower-extremity muscle strength/power were assessed in healthy individuals using the Pearson product-moment correlation coefficient (*r* value). To pool *r* values derived from different studies, “Fisher’s z’ transformation” was used, i.e., Pearson product-moment correlation coefficients were converted to the normally distributed variable z’ (i.e., *z*-transformed *r*_*z*_ value). The formula for the transformation is (Eq. 1):

1 $$ z^{\prime} = 0. 5\left[ {{ \ln }\left( { 1 + {{r}}} \right) {-} { \ln }\left( { 1 {-} {{r}}} \right)} \right] $$

where ln is the natural logarithm [29]. In addition, the included studies were weighted according to the magnitude of the respective standard error (SE). The formula for the calculation of the SE is (Eq. 2):

2 $$ {\text{SE }} = { 1}/\sqrt{ \left( {N - 3} \right)} $$

where *N* stands for the respective sample size [29]. Afterwards, weighted mean *r*_*z*_ values were computed. To classify and interpret the correlation sizes, *r*_*z*_ values were back-transformed to *r* values. Based on the recommendations of Vincent [30], values of 0.00 ≤ *r* ≤ 0.69 indicate small, 0.70 ≤ *r* ≤ 0.89 medium, and *r* ≥ 0.90 large sizes of correlation. Finally, a statistical analysis was conducted to calculate differences between the mean *r* values by age groups (children vs. young adults vs. old adults) [29, 31]. The corresponding formula is (Eq. 3):

3 $$ z = (z1 - z2)/\sqrt{(1/(n1 - 3) + 1/(n2 - 3))}. $$

## Results

### Study Characteristics

**Fig. 1:**
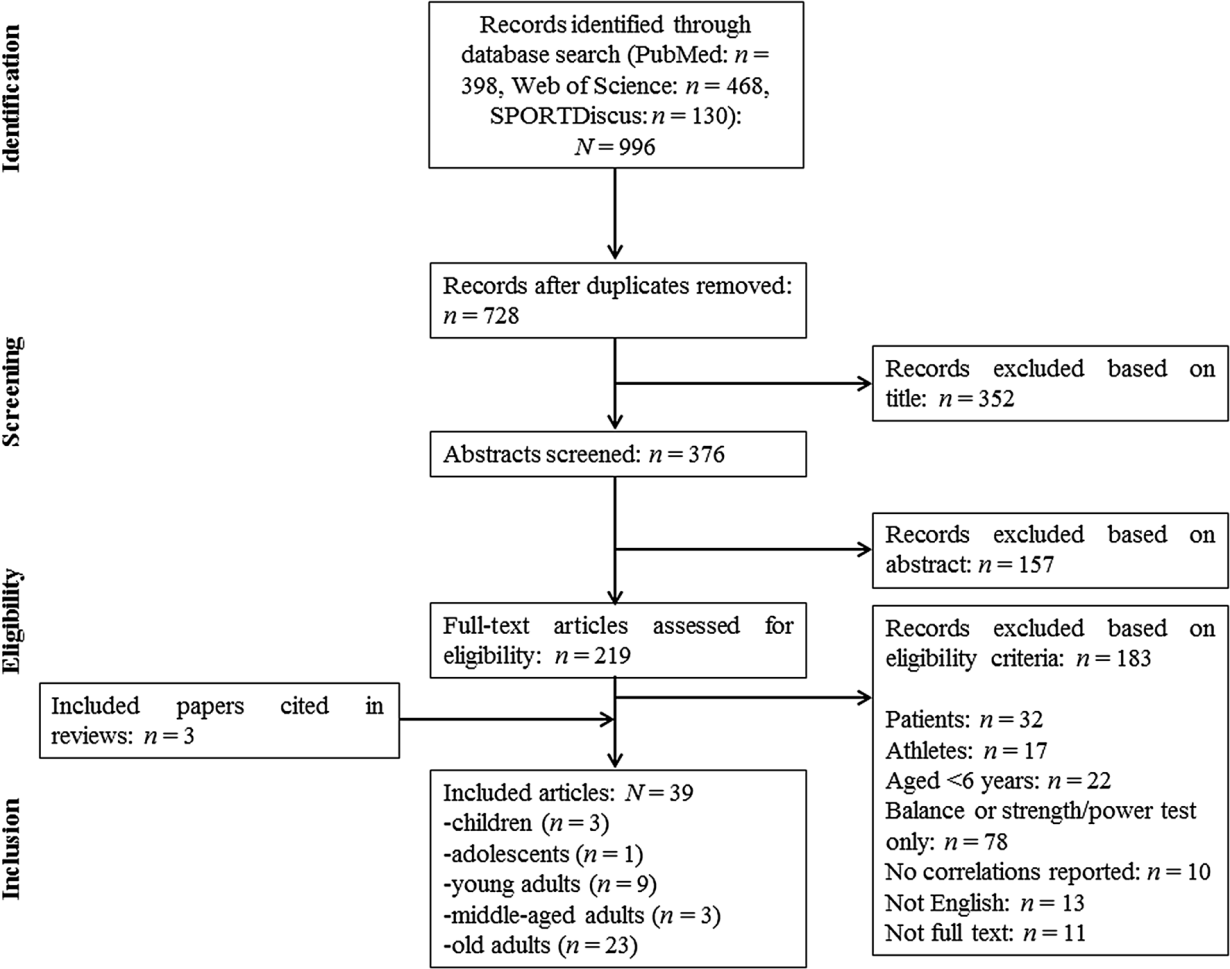
Flow chart illustrating the different phases of the search and selection

Figure 1 displays a flow chart that illustrates the different stages of the systematic search and the selection of articles over the course of the literature search. Our initial search identified 996 studies that were potentially eligible for inclusion in this systematic review. After removal of duplicates and exclusion of ineligible articles, 36 studies remained. We identified another three articles from the reference lists of the included papers and from already published review articles. Therefore, 39 studies were included in the final analysis. Table 1 illustrates the main characteristics of the included studies. Three studies examined associations of balance and lower-extremity muscle strength/power in children (*n* = 145 subjects), one study in adolescents (*n* = 28 subjects), nine studies in young adults (*n* = 285 subjects), three studies in middle-aged adults (*n* = 68 subjects), and 23 studies in old adults (*n* = 3766 subjects). All eligible studies included at least one measure of balance (e.g., CoP displacement during one-legged stance) and lower-extremity muscle strength (e.g., MVC)/power (e.g., jump height). Irrespective of the age category, 16 studies reported correlations between static steady-state balance (e.g., CoP displacement during one-legged stance) and maximal strength (e.g., MVC), four studies between static steady-state balance and explosive force (e.g., RFD), and nine studies between static steady-state balance and muscle power (e.g., jump height). In terms of dynamic steady-state balance (e.g., gait speed), 22 studies investigated correlations with maximal strength (e.g., MVC), three studies with explosive force (e.g., RFD), and nine studies with muscle power (e.g., jump height). Correlations of proactive balance (e.g., maximal reach distance in the functional-reach-test) with maximal strength (e.g., MVC) were reported in 12 studies, with explosive force (e.g., RFD) in one study, and with muscle power (e.g., jump height) in six studies. Lastly, ten studies examined correlations of reactive balance (e.g., CoP displacement during perturbed one-legged stance) with maximal strength (e.g., MVC), nine studies with explosive force (e.g., RFD), and ten studies with muscle power (e.g., jump height).

**Table 1:** Studies examining associations between measures of balance and lower-extremity muscle strength/power by age group

Study	No. of subjects; sex; age [years (range or mean ± SD)]	Balance test parameter/outcome	Strength/power test parameter/outcome	*z*-transformed *r*_*z*_ values; explained variance (*r* ^2^)
Children (*n* = 3)				
Granacher and Gollhofer [32]	30; F (16), M (14); 6–7	RB: 20-s two-legged stance after perturbation with eyes opened, CoP in ap-/ml-direction sSSB: 20-s two-legged stance with eyes opened, CoP displacements in ap-/ml-direction	MVC: ankle plantarflexor RFD: ankle plantarflexor P: CMJ, height, power	RB-MVC: 0.16, 3 % RB-RFD: 0.19, 4 % RB-P: 0.16, 3 % sSSB-MVC: 0.13, 2 % sSSB-RFD: 0.11, 1 % sSSB-P: 0.15, 2 %
Ibrahim et al. [33]	94; M; 6–10	dSSB: 20-s two-legged stance on Biodex Balance System, overall stability index	MVC: hip flexors/extensors, knee flexors/extensors, ankle dorsiflexors/plantarflexors	dSSB-MVC: 0.95, 55 %
Muehlbauer et al. [34]	21; F (8), M (13); 7–10	dSSB: 10-m walk, speed PB: TUG, time; FRT, distance RB: 10-s two-legged stance after perturbation with eyes opened, SO in ap-/ml-direction sSSB: 30-s two-legged stance with eyes opened, CoP displacements in ap-/ml-direction	MVC: leg extensors P: CMJ, height	dSSB-MVC: 0.28, 7 % dSSB-P: 0.46, 18 % PB-MVC: TUG = 0.42, 16 %; FRT = 0.61, 30 % PB-P: TUG = 0.45, 18 %; FRT = 0.44, 17 % RB-MVC: 0.15, 2 % RB-P: 0.17, 3 % sSSB-MVC: 0.09, 1 % sSSB-P: 0.31, 9 %
Adolescents (*n* = 1)				
Granacher and Gollhofer [35]	28; F (15), M (13); 16–17	RB: 10-s one-legged stance after perturbation with eyes opened, SO in ap-/ml-direction sSSB: 30-s one-legged stance with eyes opened, CoP displacements in ap-/ml-direction	MVC: leg extensors RFD: leg extensors P: CMJ, force, height	RB-MVC: 0.07, 0 % RB-RFD: 0.09, 1 % RB-P: 0.12, 1 % sSSB-MVC: 0.08, 1 % sSSB-RFD: 0.07, 0 % sSSB-P: 0.11, 1 %
Young adults (*n* = 9)				
Izquierdo et al. [37]	12; M; 21 ± 1	dSSB: alternating knee raise, total CoP displacement area, length RB: 30-s two-legged stance with eyes opened and to score a target in response to an external signal, time to reach/remain in the target	MVC: leg extensors RFD: leg extensors P: CMJ, height; SJ, height; SLJ, distance	dSSB-MVC: 0.10, 1 % dSSB-RFD: 0.34, 11 % dSSB-P: 0.31, 9 % RB-MVC: 0.25, 6 % RB-RFD: 0.18, 3 % RB-P: 0.30, 8 %
Katayama et al. [38]	57; F; 23 ± 2	sSSB: 30-s two-legged stance with eyes opened/closed; 10-s one-legged stance with eyes opened/closed, total CoP displacement area, length	P: knee flexors/extensors	sSSB-P: 0.29, 8 %
McCurdy and Langford [39]	42; F (25), M (17); 22 ± 2	dSSB: one-legged stance with eyes opened on unstable ground; time sSSB: one-legged stance with eyes opened, time	MVC: 1RM unilateral squat	sSSB-MVC: 0.21, 4 % dSSB-MVC: 0.09, 1 %
Oshita and Yano [40]	14; M; 21 ± 1	sSSB: one-legged stance with eyes closed, time	MVC: ankle plantarflexors	sSSB-MVC: 0.13, 2 %
Piirainen et al. [42]	10; M; 27 ± 3	RB: 30-s two-legged stance following a perturbation impulse with eyes opened, CoP displacements in ap-/ml-direction sSSB: 5-s two-legged stance with eyes opened, CoP displacements in ap-/ml-direction	MVC: knee extensors, ankle plantarflexors RFD: knee extensors, ankle plantarflexors	RB-MVC: NR RB-RFD: 0.27, 7 % sSSB-MVC: NR sSSB-RFD: NR
Oshita and Yano [41]	12; M; 21 ± 1	sSSB: two-legged stance with eyes opened, total CoP displacement velocity	MVC: ankle plantarflexors	sSSB-MVC: 0.33, 10 %
Hesari et al. [44]	47; F (24), M (23); 20 ± 4	PB: star-excursion-balance test	MVC: hip flexors/extensors/abductors/adductors	PB-MVC: 0.29, 8 %
Muehlbauer et al. [43]	27; F (19), M (8); 23 ± 4	RB: 10-s one-legged stance following a perturbation impulse with eyes opened, CoP displacements in ap-/ml-direction sSSB: 30-s one-legged stance with eyes opened, CoP displacements in ap-/ml-direction	MVC: ankle plantarflexors RFD: ankle plantarflexors P: CMJ, height, power	RB-MVC: 0.24, 6 % RB-RFD: 0.30, 8 % RB-P: 0.12, 1 % sSSB-MVC: 0.16, 3 % sSSB-RFD: 0.05, 0 % sSSB-P: 0.09, 1 %
Teyhen et al. [36]	64; F (11), M (53); 25 ± 4	PB: Y-balance test, reach distance	MVC: hip external rotators/abductors P: 6-m timed hop test, time; triple crossover hop test, distance	RB-MVC: NR RB-P: 0.35, 11 %
Middle-aged adults (*n* = 3)				
Izquierdo et al. [37]	10; M; 40 ± 2	dSSB: alternating knee raise, total CoP displacement area, length RB: 30-s two-legged stance with eyes opened and to score a target in response to an external signal, time to reach/remain in the target	MVC: leg extensors RFD: leg extensors P: CMJ, height; SJ, height; SLJ, distance	dSSB-MVC: 0.09, 1 % dSSB-RFD: 0.30, 8 % dSSB-P: 0.38, 13 % RB-MVC: 0.38, 13 % RB-RFD: 0.77, 42 % RB-P: 0.22, 5 %
Holviala et al. [46]	26; F; 53 ± 2	dSSB: 10-m walk, time	MVC: leg extensors	dSSB-MVC: 0.69, 36 %
Muehlbauer et al. [45]	32; F (9), M (23); 56 ± 4	RB: 10-s one-legged stance following a perturbation impulse with eyes opened, CoP displacements in ap-/ml-direction sSSB: 30-s one-legged stance with eyes opened, CoP displacements in ap-/ml-direction	MVC: ankle plantarflexors RFD: ankle plantarflexors P: CMJ, height, power	RB-MVC: 0.09, 1 % RB-RFD: 0.10, 1 % RB-P: 0.09, 1 % sSSB-MVC: 0.09, 1 % sSSB-RFD: 0.15, 2 % sSSB-P: 0.24, 6 %
Old adults (*n* = 23)				
Iverson et al. [47]	54; M; 60–90	sSSB: sharpened Romberg test, time; one-legged stance test with eyes opened/closed, time	MVC: hip flexors/extensors/abductors	sSSB-MVC: 0.42, 16 %
Buchner et al. [48]	409; F (245), M (164); 60–96	dSSB: 15.2-m walk, gait speed	MVC: knee extensors/flexors, ankle dorsiflexors/plantarflexors	dSSB-MVC: 0.45, 18 %
Judge et al. [49]	26; NR; 79 ± 6	dSSB: 5-m walk, step length	MVC: knee extensors, ankle plantarflexors	dSSB-MVC: 0.62, 30 %
Izquierdo et al. [37]	10; M; 71 ± 5	dSSB: alternating knee raise, total CoP displacement area, length RB: 30-s two-legged stance with eyes opened and to score a target in response to an external signal, time to reach/remain in the target	MVC: leg extensors RFD: leg extensors P: CMJ, height; SJ, height; SLJ, distance	dSSB-MVC: 0.07, 0 % dSSB-RFD: 0.22, 5 % dSSB-P: 0.42, 16 % RB-MVC: 0.19, 4 % RB-RFD: 0.37, 13 % RB-P: 0.43, 16 %
Ringsberg et al. [50]	230; F; 75	dSSB: 30-m walk, time, cadence RB: 20-s two-legged stance with eyes opened on a moving platform, total CoP displacement length sSSB: one-legged stance, time; 20-s two-legged stance with eyes opened/closed, total CoP displacement length	MVC: knee extensors/flexors, ankle dorsiflexors	dSSB-MVC: 0.34, 11 % RB-MVC: NR sSSB-MVC: 0.19, 4 %
Burnfield et al. [51]	81; M; 60–92	dSSB: 10-m walk, gait speed	MVC: hip extensors/flexors, knee extensors/flexors, ankle dorsiflexors/plantarflexors	dSSB-MVC: 0.55, 25 %
Suzuki et al. [52]	34; F; 65–84	dSSB: 10-m forward/backward tandem walk at habitual/maximal speed, time	MVC: ankle plantarflexors/dorsiflexors P: ankle plantarflexors/dorsiflexors	dSSB-MVC: 0.46, 18 % dSSB-P: 0.47, 19 %
Menz et al. [53]	176; F (120), M (56); 62–96	dSSB: 6-m walk, gait speed PB: alternate step test, time sSSB: two-legged stance with eyes opened, total CoP displacement area, length	MVC: knee extensors, ankle dorsiflexors	dSSB-MVC: 0.64, 32 % PB-MVC: 0.56, 26 % sSSB-MVC: 0.34, 11 %
Tsang and Hui-Chan [65]	48; F (24), M (24); 70 ± 5	sSSB: 30-s two-legged stance with eyes opened, total CoP displacement angle RB: one-legged stance while perturbation with eyes opened, CoP displacement angle	MIT: knee flexors/extensors	sSSB-MIT: 0.07, 0 % RB-MIT: 0.47, 19 %
Callisaya et al. [64]	278; F (124), M (154); 60–86	dSSB: 4.6-m walk, speed, cadence, step length/width sSSB: 30-s two-legged stance with eyes closed/opened, total CoP displacement length	MVC: leg extensors	dSSB-MVC: 0.28, 7 % sSSB-MVC: NR
Melzer et al. [54]	43, F (27), M (16);78 ± 6	PB: LOS test, total CoP displacement length sSSB: 30-s two-legged stance with eyes opened, total CoP displacement area, length, velocity	MVC: ankle dorsiflexors/plantarflexors	PB-MVC: 0.46, 18 % sSSB-MVC: 0.08, 1 %
Piirainen et al. [42]	20; M; 64 ± 3	RB: 30-s two-legged stance while perturbation with eyes opened, CoP displacements in ap-/ml-direction sSSB: 5-s two-legged stance with eyes opened, CoP displacements in ap-/ml-direction	MVC: knee extensors, ankle plantarflexors RFD: knee extensors, ankle plantarflexors	RB-MVC: NR RB-RFD: 0.54, 24 % sSSB-MVC: NR sSSB-RFD: NR
Bouchard et al. [55]	1,280, F (645), M (635);68 ± 9	dSSB: 20-foot walk, gait speed	MVC: leg extensors	dSSB-MVC: 0.24, 6 %
Shimada et al. [56]	213; F (130), M (83); 65–96	dSSB: 6-m walk, time PB: TUG, time sSSB: one-legged stance with eyes opened, time	P: CST, time	dSSB-P: 0.39, 14 % PB-P: 0.41, 15 % sSSB-P: 0.05, 0 %
Spink et al. [57]	305; 211 (F), M (94); 65–93	dSSB: 6-m walk, gait speed PB: alternate step test, time sSSB: two-legged stance with eyes opened, total CoP displacement area, length	MVC: ankle plantarflexors/dorsiflexors/inversors/eversors P: CST, time	dSSB-MVC: 0.22, 5 % dSSB-P: NR PB-MVC: 0.38, 13 % PB-P: NR sSSB-MVC: 0.19, 4 % sSSB-P: NR
Marcus et al. [58]	109; F (77), M (32); 74 ± 7	PB: TUG, time	MVC: knee extensors	PB-MVC: 0.48, 20 %
Muehlbauer et al. [59]	24; F (13), M (11); 70 ± 5	dSSB: 10-m walk, gait speed PB: TUG, time; FRT, distance RB: 10-s two-legged stance following a perturbation impulse with eyes opened, SO in ap-/ml-direction sSSB: 30-s two-legged stance with eyes opened, CoP displacements in ap-/ml-direction	MVC: leg extensors P: CMJ, height, power	dSSB-MVC: 0.03, 0 % dSSB-P: 0.17, 3 % PB-MVC: TUG = 0.10, 1 %; FRT = 0.51, 22 % PB-P: TUG = 0.36, 12 %; FRT = 0.21, 4 % RB-MVC: 0.15, 2 % RB-P: 0.08, 1 % sSSB-MVC: 0.32, 10 % sSSB-P: 0.23, 5 %
Shin et al. [60]	72; F (43), M (29); 70 ± 6	dSSB: 8.3-m walk, stride length/time variability	MVC: leg extensors/flexors, ankle dorsiflexors/plantarflexors	dSSB-MVC: 0.07, 0 %
Forte et al. [27]	57; F (33), M (24); 70 ± 3	dSSB: 10-m walk, gait speed	MVC: knee extensors P: CMJ, power	dSSB-MVC: 0.37, 13 % dSSB-P: 0.35, 11 %
Miyazaki et al. [61]	124; M; 73 ± 7	dSSB: 11-m walk, gait speed PB: TUG, time sSSB: one-legged stance with eyes opened, time	MVC: leg extensors	dSSB-MVC: 0.60, 29 % PB-MVC: 0.69, 36 % sSSB-MVC: 0.59, 28 %
Forte et al. [26]	57; F (33), M (24); 65–75	dSSB: 10-m walk, gait speed sSSB: Romberg test, total CoP displacement length, velocity; two-legged tandem stance test with eyes opened, total CoP displacement length, velocity	MVC: knee extensors P: CMJ, power	dSSB-MVC: 0.28, 7 % dSSB-P: 0.27, 7 % sSSB-MVC: 0.35, 11 % sSSB-P: 0.33, 10 %
Jenkins et al. [62]	16; M; 72 ± 7	PB: FRT, distance	MVC: leg extensors RTD: leg extensors at 60–180 °/s P: leg extensors	PB-MVC: 0.23, 5 % PB-RTD: 0.22, 5 % PB-P: 0.54, 24 %
Pisciottano et al. [63]	100; F; 71 ± 5	PB: TUG, time	MVC: knee extensors/flexors	PB-MVC: 0.30, 8 %

*1RM* one-repetition maximum, *ap* anterior-posterior, *CMJ* countermovement jump, *CoP* center of pressure, *CST* chair-stand-test, *dSSB* dynamic steady-state balance, *F* female, *FRT* functional-reach-test, *LOS* limits-of-stability-test, *M* male, *ml* medio-lateral, *MIT* maximum isokinetic torque, *MVC* maximum voluntary contraction, *NR* not reported, *P* Power, *PB* proactive balance, *RB* reactive balance, *RFD* rate of force development, *RTD* rate of torque development, *r*_*z*_ weighted *z*-transformed Pearson’s correlation coefficients, *SD* standard deviation, *sSSB* static steady-state balance, *SJ* squat jump, *SLJ* standing long jump, *SO* summed oscillations, *TUG* timed-up-and-go-test

### Associations Between Measures of Balance and Lower-Extremity Muscle Strength/Power

#### Children

**Fig. 2:**
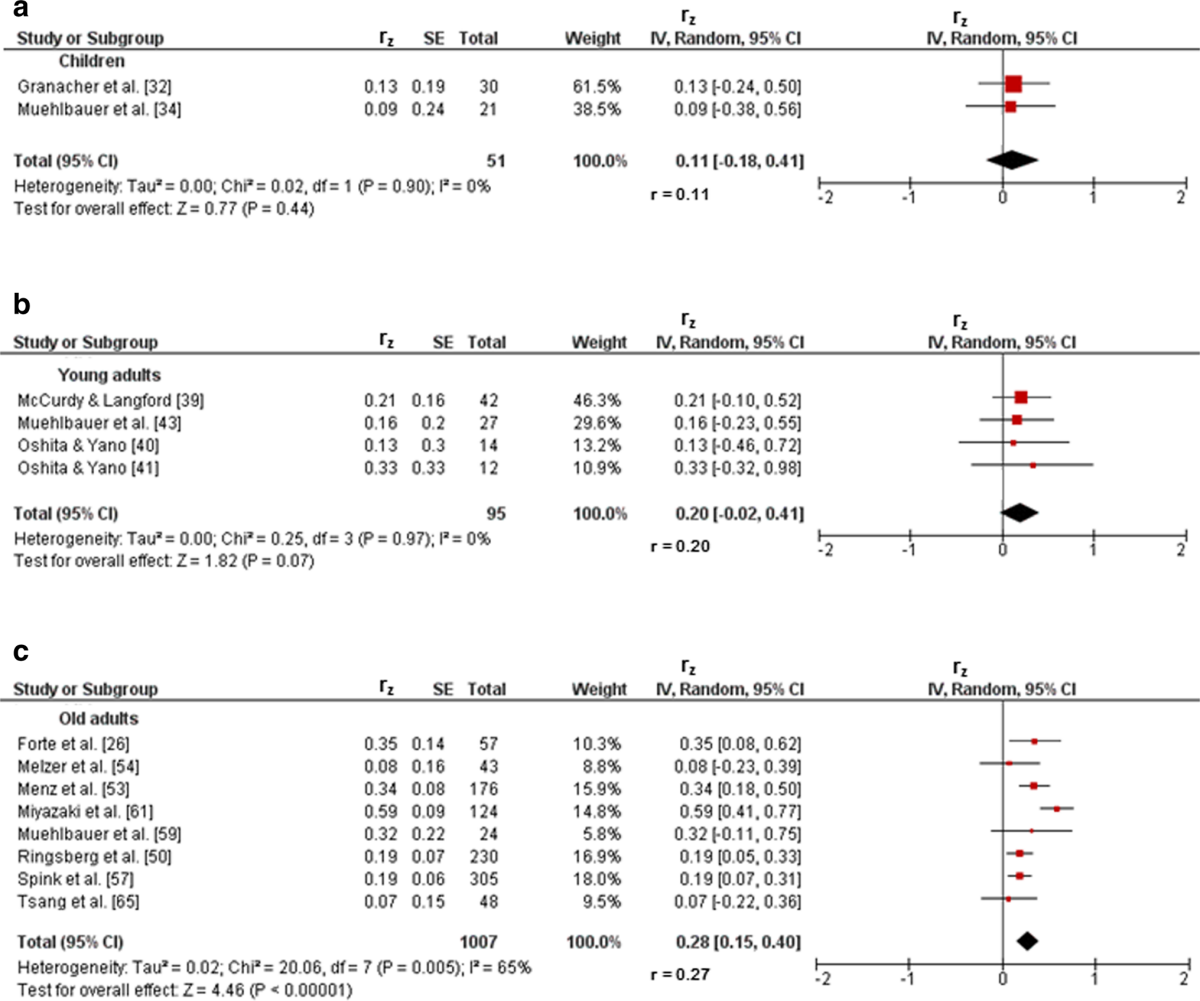
Associations between static steady-state balance (e.g., postural sway during one-legged stance) and maximal strength (e.g., maximum voluntary contraction) of the lower-extremities in children (**a**), young adults (**b**), and old adults (**c**). *CI* confidence interval, *df* degrees of freedom, *IV* inverse variance, *r* back-transformed Pearson’s correlation coefficients, *r*_*z*_ weighted *z*-transformed Pearson’s correlation coefficients, *SE* standard error

**Fig. 3:**
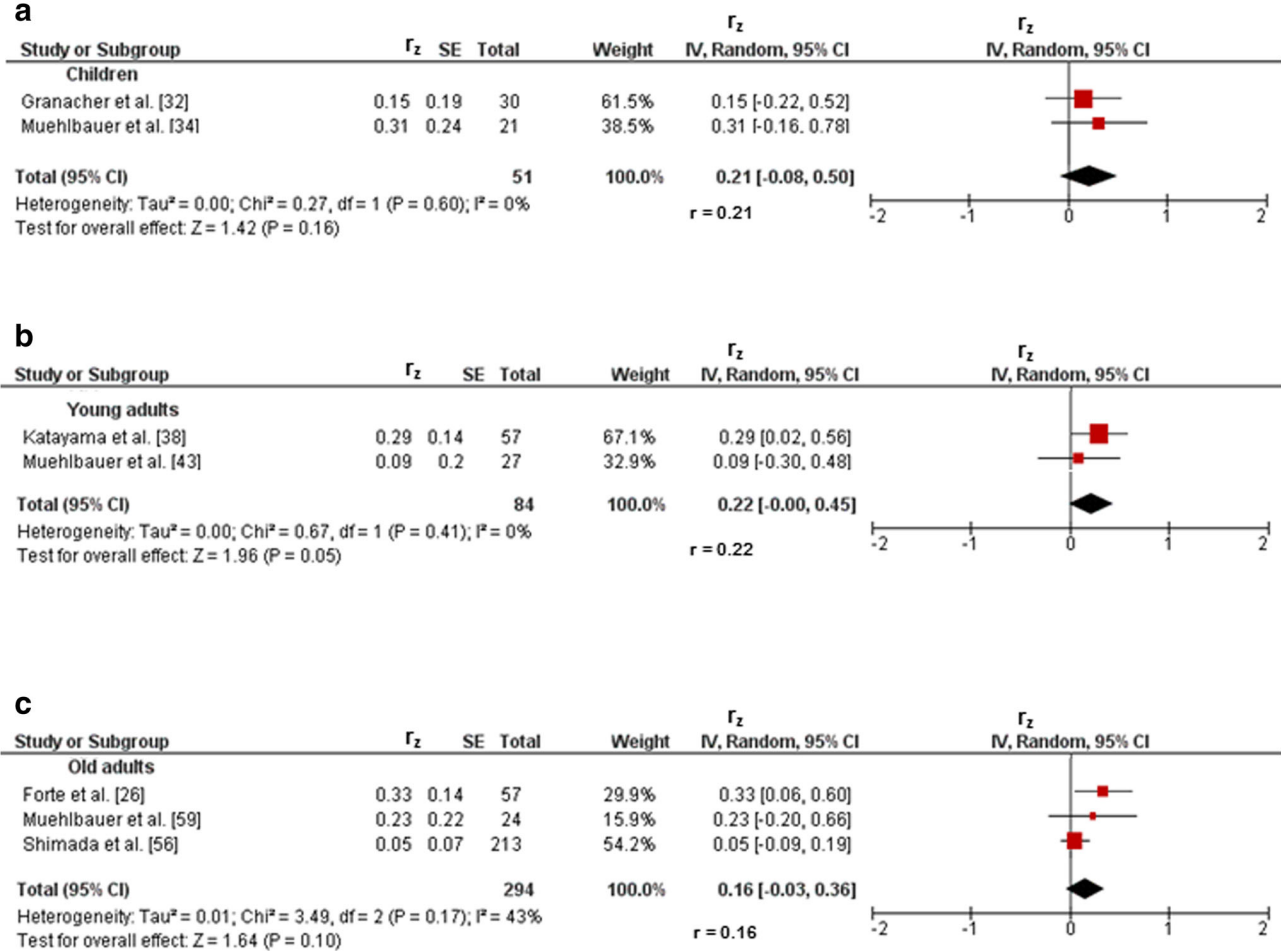
Pearson’s *r* values (*z*-transformed) for associations between static steady-state balance (e.g., postural sway during one-legged stance) and muscle power (e.g., jump height) of the lower-extremities in children (**a**), young adults (**b**), and old adults (**c**). *CI* confidence interval, *df* degrees of freedom, *IV* inverse variance, *r* back-transformed Pearson’s correlation coefficients, *r*_*z*_ weighted *z*-transformed Pearson’s correlation coefficients, *SE* standard error

**Fig. 4:**
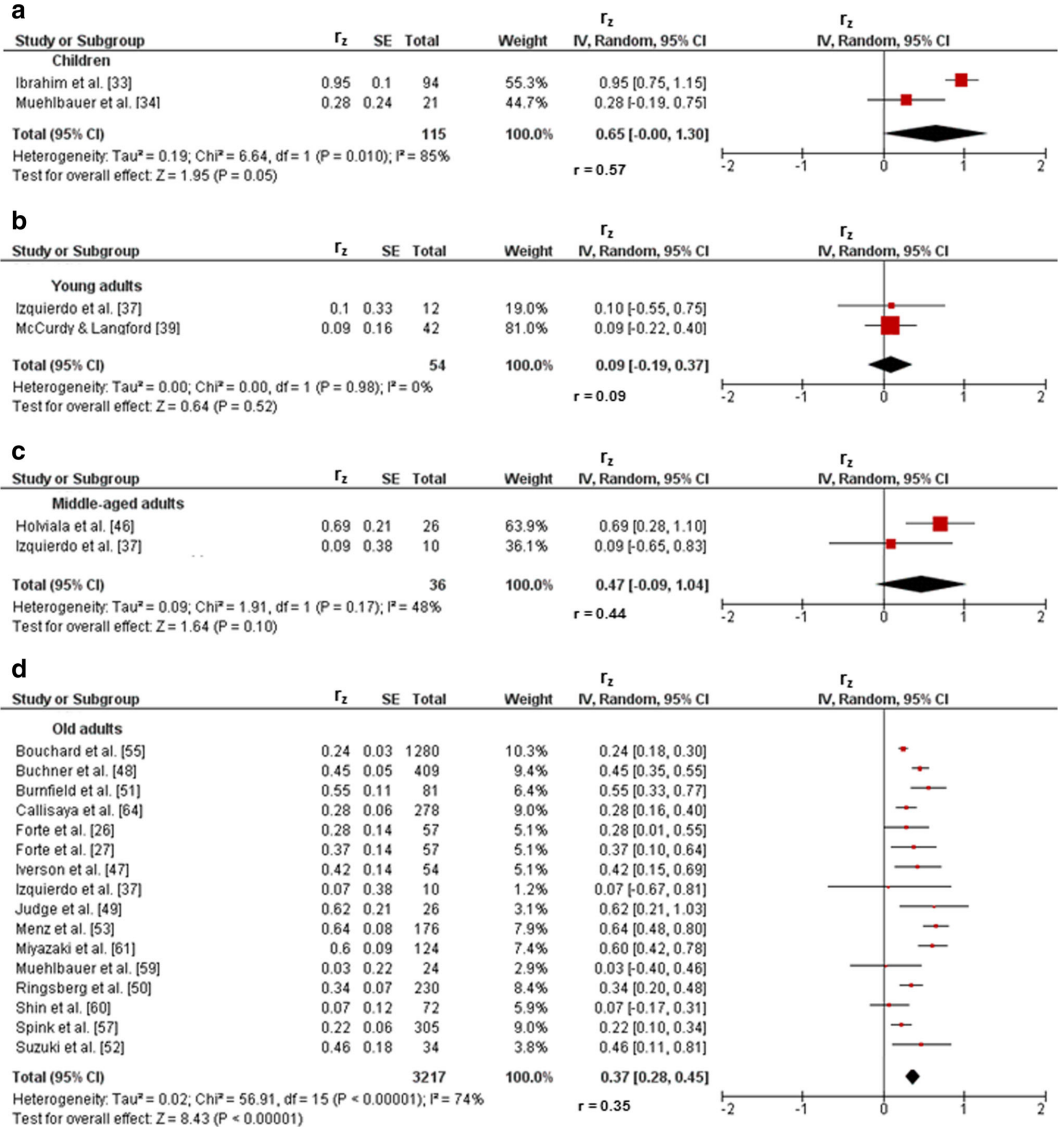
Pearson’s *r* values (*z*-transformed) for associations between dynamic steady-state balance (e.g., gait speed) and maximal strength (e.g., maximum voluntary contraction) of the lower-extremities in children (**a**), and young (**b**), middle-aged (**c**), and old (**d**) adults. *CI* confidence interval, *df* degrees of freedom, *IV* inverse variance, *r* back-transformed Pearson’s correlation coefficients, *r*_*z*_ weighted *z*-transformed Pearson’s correlation coefficients, *SE* standard error

**Fig. 5:**
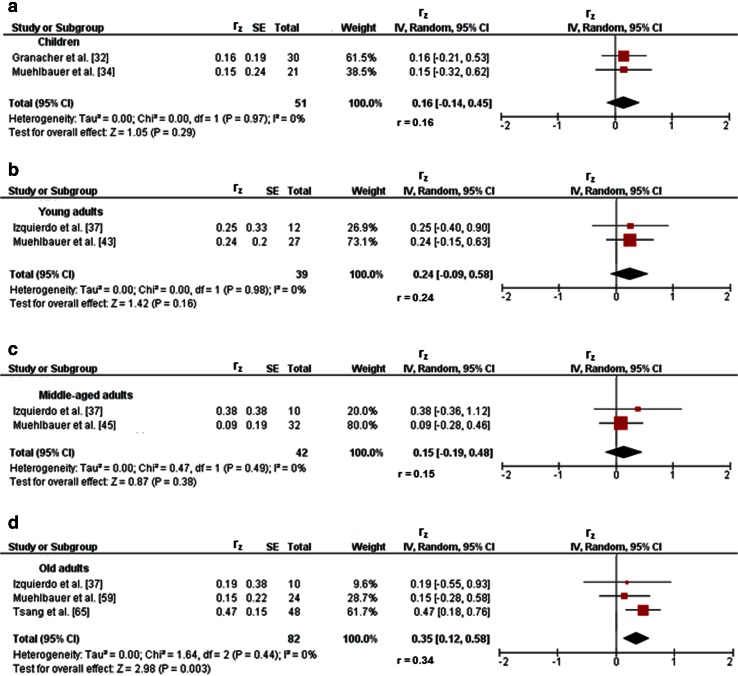
Pearson’s *r* values (*z*-transformed) for associations between reactive balance (e.g., postural sway during perturbed one-legged stance) and maximal strength (e.g., maximum voluntary contraction) of the lower-extremities in children (**a**), and young (**b**), middle-aged (**c**), and old (**d**) adults. *CI* confidence interval, *df* degrees of freedom, *IV* inverse variance, *r* back-transformed Pearson’s correlation coefficients, *r*_*z*_ weighted *z*-transformed Pearson’s correlation coefficients, *SE* standard error

**Fig. 6:**
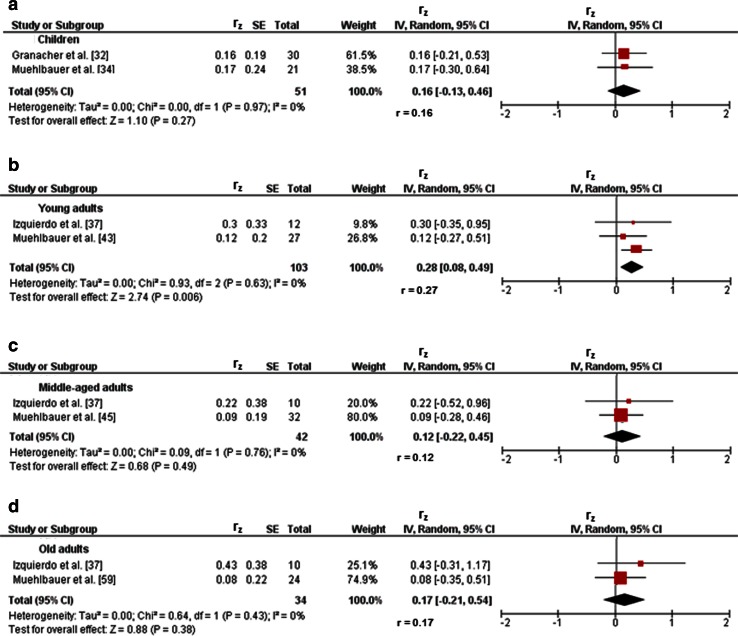
Pearson’s *r* values (*z*-transformed) for associations between reactive balance (e.g., postural sway during perturbed one-legged stance) and muscle power (e.g., jump height) of the lower-extremities in children (**a**), and young (**b**), middle-aged (**c**), and old (**d**) adults. *CI* confidence interval, *df* degrees of freedom, *IV* inverse variance, *r* back-transformed Pearson’s correlation coefficients, *r*_*z*_ weighted *z*-transformed Pearson’s correlation coefficients, *SE* standard error

Three studies investigated associations between variables of balance and lower-extremity muscle strength/power in children [32–34]. Figures 2a and 3a illustrate the associations of measures of static steady-state balance with maximal strength and muscle power. Weighted mean *r*_z_ values amounted to 0.11 for measures of maximal strength (*I*^2^ = 0 %, Chi-squared (*χ*^2^) = 0.02, degrees of freedom [*df*] = 1, *p* = 0.90, two studies [32, 34]) and 0.21 for outcomes of muscle power (*I*^2^ = 0 %, *χ*^2^ = 0.27, *df* = 1, *p* = 0.60, two studies [32, 34]). Back-transformed *r* values of 0.11 and 0.21 indicated small-sized correlations. Only one study [32] reported a small correlation (*r*_z_ = 0.11, *r* = 0.11) between static steady-state balance (i.e., 20-s two-legged stance) and explosive force (i.e., RFD ankle plantarflexors) (Table 1). Additionally, associations between dynamic steady-state balance and maximal strength revealed a weighted mean *r*_z_ value of 0.65 (*I*^2^ = 85 %, *χ*^2^ = 6.64, *df* = 1, *p* = 0.01, two studies [33, 34], Fig. 4a). The corresponding back-transformed *r* value of 0.57 is indicative of small-sized correlations. No study reported associations of dynamic steady-state balance with explosive force and muscle power. Only one study [34] observed small associations between proactive balance (i.e., FRT) and maximal strength (i.e., MVC leg extensors) (*r*_z_ = 0.61, *r* = 0.54) and muscle power (i.e., CMJ) (*r*_z_ = 0.44, *r* = 0.41) (Table 1). No study reported associations of proactive balance with explosive force. Lastly, associations of reactive balance with maximal strength and muscle power are shown in Figs. 5a and 6a, respectively. Weighted mean *r*_z_ values amounted to 0.16 for measures of maximal strength (*I*^2^ = 0 %, *χ*^2^ = 0, *df* = 1, *p* = 0.97, two studies [32, 34]) and 0.16 for outcomes of muscle power (*I*^2^ = 0 %, *χ*^2^ = 0, *df* = 1, *p* = .97, two studies [32, 34]). Back-transformed *r* values of 0.16 and 0.16 indicated small-sized correlations. In addition, one study [32] observed a small association (*r*_z_ = 0.19, *r* = 0.19) between measures of reactive balance (i.e., perturbed 20-s two-legged stance) and explosive force (i.e., CMJ) (Table 1).

#### Adolescents

Only one study [35] analyzed associations between measures of balance and muscle strength/power of the lower-extremities in adolescents. As a result, small-sized correlations were detected for measures of static steady-state balance (i.e., 30-s one-legged stance) with maximal strength (i.e., MVC leg extensors) (*r*_*z*_ = 0.08, *r* = 0.08), explosive force (i.e., RFD leg extensors) (*r*_*z*_ = 0.07, *r* = 0.07), and muscle power (i.e., CMJ) (*r*_*z*_ = 0.11, *r* = 0.11) (Table 1). In addition, small correlations were obtained for reactive balance (i.e., perturbed 10-s one legged stance) with maximal strength (*r*_*z*_ = 0.07, *r* = 0.07), explosive force (*r*_*z*_ = 0.09, *r* = 0.09), and muscle power (*r*_*z*_ = 0.12, *r* = 0.12) (Table 1). No study reported associations for measures of dynamic steady-state and proactive balance with maximal strength, explosive force, and muscle power.

#### Young Adults

**Fig. 7:**
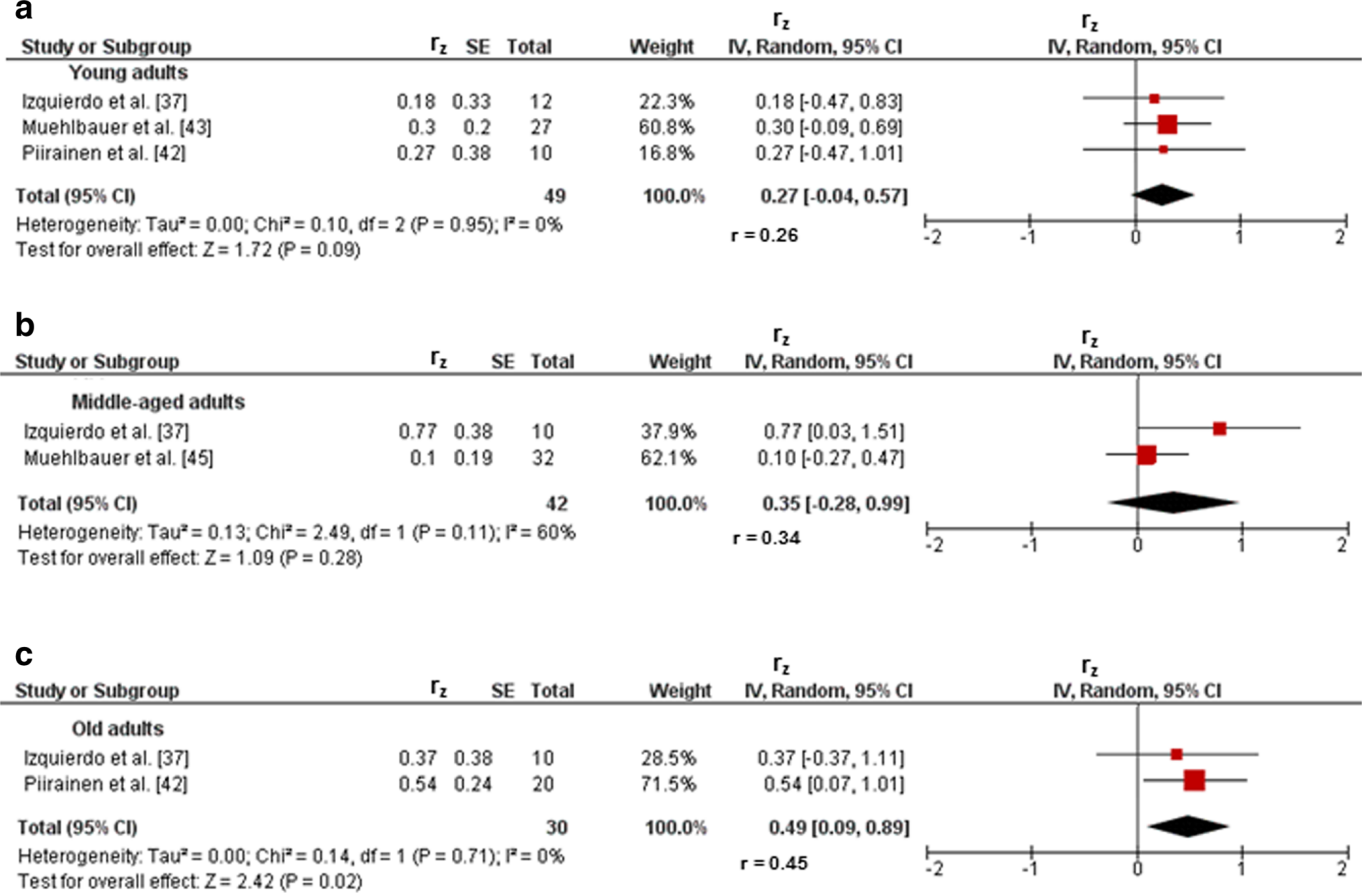
Pearson’s *r* values (*z*-transformed) for associations between reactive balance (e.g., postural sway during perturbed one-legged stance) and explosive force (e.g., rate of force development) of the lower-extremities in young (**a**), middle-aged (**b**), and old adults (**c**). *CI* confidence interval, *df* degrees of freedom, *IV* inverse variance, *r* back-transformed Pearson’s correlation coefficients, *r*_*z*_ weighted *z*-transformed Pearson’s correlation coefficients, *SE* standard error

Nine studies reported associations between proxies of balance and lower-extremity muscle strength/power in young adults [36–44]. Figures 2b and 3b illustrate associations of static steady-state balance with maximal strength and muscle power, respectively. Weighted mean *r*_z_ values amounted to 0.20 for variables of maximal strength (*I*^2^ = 0 %, *χ*^2^ = 0.25, *df* = 3, *p* = 0.97, four studies [39–41, 43]) and 0.22 for measures of muscle power (*I*^2^ = 0 %, *χ*^2^ = 0.67, *df* = 1, *p* = 0.41, two studies [38, 43]). Back-transformed *r* values of 0.20 and 0.22 indicated small-sized correlations. Only one study [43] observed a small-sized correlation (*r*_*z*_ = 0.05, *r* = 0.05) between static steady-state balance (i.e., 30-s one-legged stance) and explosive force (i.e., CMJ) (Table 1). In addition, associations between dynamic steady-state balance and maximal strength resulted in a weighted mean *r*_z_ value of 0.09 (*I*^2^ = 0 %, *χ*^2^ = 0, *df* = 1, *p* = 0.98, two studies [37, 39], Fig. 4b). The corresponding back-transformed *r* value of 0.09 is indicative of small-sized correlations. Only one study [37] reported small-sized associations of dynamic steady-state balance (i.e., alternating knee raise) with explosive force (i.e., RFD leg extensors) (*r*_z_ = 0.34, *r* = 0.33) and muscle power (i.e., CMJ) (*r*_z_ = 0.31, *r* = 0.30) (Table 1). Furthermore, only one study [44] stated a small-sized relationship (*r*_z_ = 0.29, *r* = 0.28) between proactive balance (i.e., maximal reach distance in the star-excursion-balance-test) and maximal strength (i.e., MVC) (Table 1). Yet, no study reported associations of proactive balance with explosive force and muscle power. Lastly, associations of reactive balance with maximal strength, explosive force, and muscle power are illustrated in Figs. 5b, 6b, and 7a, respectively. Weighted mean *r*_z_ values amounted to 0.24 for measures of maximal strength (*I*^2^ = 0 %, *χ*^2^ = 0, *df* = 1, *p* = 0.98, two studies [37, 43]), 0.27 for variables of explosive force (*I*^2^ = 0 %, *χ*^2^ = 0.10, *df* = 2, *p* = 0.95, three studies [37, 42, 43]), and 0.28 for outcomes of muscle power (*I*^2^ = 0 %, *χ*^2^ = 0.93, *df* = 2, *p* = 0.63, three studies [36, 37, 43]). Back-transformed *r* values of 0.24, 0.26, and 0.27 indicated small-sized correlations.

#### Middle-Aged Adults

Three studies provided associations between outcomes of balance and muscle strength/power of the lower-extremities in middle-aged adults [37, 45, 46]. Only one study [45] reported small-sized correlations of static steady-state balance (i.e., 30-s one-legged stance) with maximal strength (i.e., MVC ankle plantarflexors) (*r*_z_ = 0.09, *r* = 0.09), explosive force (i.e., RFD ankle plantarflexors) (*r*_z_ = 0.15, *r* = 0.15), and muscle power (i.e., CMJ) (*r*_z_ = 0.24, *r* = 0.24) (Table 1). Additionally, associations between dynamic steady-state balance and maximal strength revealed a weighted mean *r*_z_ value of 0.47 (*I*^2^ = 48 %, *χ*^2^ = 1.91, *df* = 1, *p* = 0.17, two studies [37, 46], Fig. 4c). The corresponding back-transformed *r* value of 0.44 is indicative of small-sized correlations. Additional small associations of dynamic steady-state balance (i.e., alternating knee raise) with explosive force (i.e., RFD leg extensors) (*r*_z_ = 0.30, *r* = 0.29) and muscle power (i.e., CMJ) (*r*_z_ = 0.38, *r* = 0.36) were observed in one study only [37] (Table 1). No study reported associations of proactive balance with maximal strength, explosive force, and muscle power. Furthermore, associations of reactive balance with maximal strength, explosive strength, and muscle power are shown in Figs. 5c, 6c, and 7b, respectively. Weighted mean *r*_z_ values amounted to 0.15 for measures of maximal strength (*I*^2^ = 0 %, *χ*^2^ = 0.47, *df* = 1, *p* = 0.49, two studies [37, 45]), 0.35 for variables of explosive force (*I*^2^ = 60 %, *χ*^2^ = 2.49, *df* = 1, *p* = 0.11, two studies [37, 45]), and 0.12 for outcomes of muscle power (*I*^2^ = 0 %, *χ*^2^ = 0.09, *df* = 1, *p* = 0.76, two studies [37, 45]). Back-transformed *r* values of 0.15, 0.34, and 0.12 indicated small-sized correlations.

#### Old Adults

**Fig. 8:**
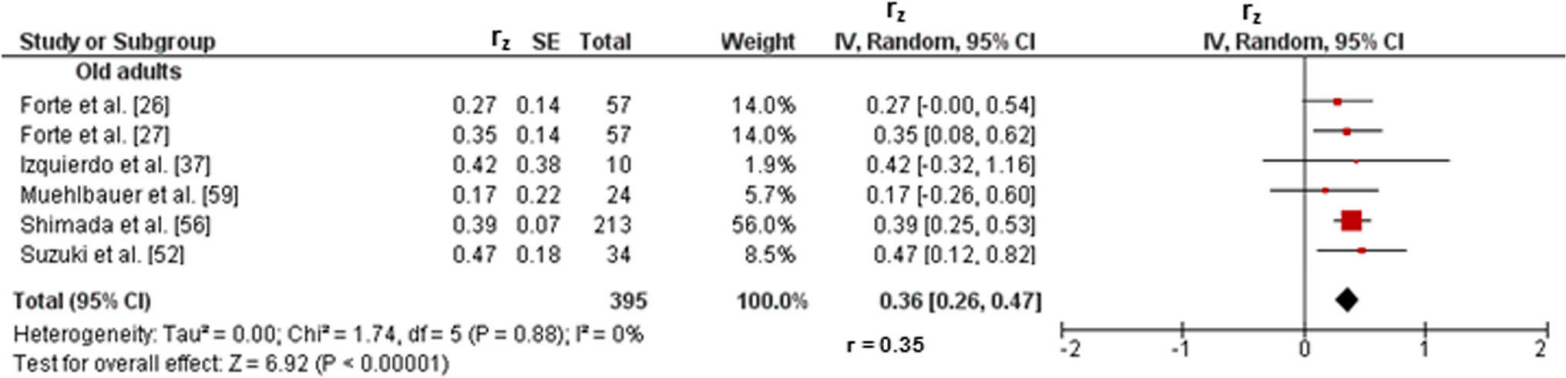
Pearson’s *r* values (*z*-transformed) for associations between dynamic steady-state balance (e.g., gait speed) and muscle power (e.g., jump height) of the lower-extremities in old adults. *CI* confidence interval, *df* degrees of freedom, *IV* inverse variance, *r* back-transformed Pearson’s correlation coefficients, *r*_*z*_ weighted *z*-transformed Pearson’s correlation coefficients, *SE* standard error

**Fig. 9:**
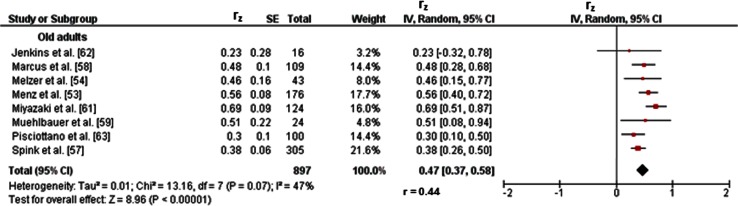
Pearson’s *r* values (*z*-transformed) for associations between proactive balance (e.g., distance in the functional-reach-test) and maximal strength (e.g., maximum voluntary contraction) of the lower-extremities in old adults. *CI* confidence interval, *df* degrees of freedom, *IV* inverse variance, *r* back-transformed Pearson’s correlation coefficients, *r*_*z*_ weighted *z*-transformed Pearson’s correlation coefficients, *SE* standard error

**Fig. 10:**
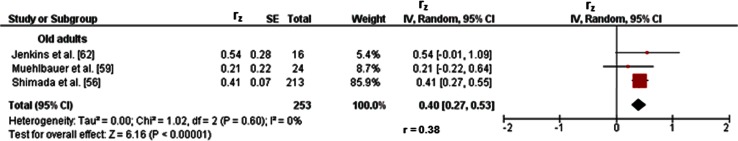
Pearson’s *r* values (*z*-transformed) for associations between proactive balance (e.g., distance in the functional-reach-test) and muscle power (e.g., jump height) of the lower-extremities in old adults. *CI* confidence interval, *df* degrees of freedom, *IV* inverse variance, *r* back-transformed Pearson’s correlation coefficients, *r*_*z*_ weighted *z*-transformed Pearson’s correlation coefficients, *SE* standard error

Twenty-three studies reported associations between parameters of balance and lower-extremity muscle strength/power in old adults [26, 27, 37, 42, 47–65]. Figures 2c and 3c illustrate the associations of static steady-state balance with maximal strength and muscle power, respectively. Weighted mean *r*_z_ values amounted to 0.28 for measures of maximal strength (*I*^2^ = 65 %, *χ*^2^ = 20.06, *df* = 7, *p* = 0.005, eight studies [26, 50, 53, 54, 57, 59, 61, 65]) and 0.16 for outcomes of muscle power (*I*^2^ = 43 %, *χ*^2^ = 3.49, *df* = 2, *p* = 0.17, three studies [26, 56, 59]). Back-transformed *r* values of 0.27 and 0.16 indicated small-sized correlations. No study reported associations between static steady-state balance and explosive force. Further associations of dynamic steady-state balance with maximal strength and muscle power are shown in Figs. 4d and 8, respectively. Weighted mean *r*_z_ values amounted to 0.37 for measures of maximal strength (*I*^2^ = 74 %, *χ*^2^ = 56.91 *df* = 15, *p* < 0.001, 16 studies [26, 27, 37, 47–53, 55, 57, 59–61, 64]) and 0.36 for outcomes of muscle power (*I*^2^ = 0 %, *χ*^2^ = 1.74, *df* = 5, *p* = 0.88, six studies [26, 27, 37, 52, 56, 59]). Back-transformed *r* values of 0.35 and 0.35 indicated small-sized correlations. Only one study [37] reported a small-sized association (*r*_z_ = 0.22, *r* = 0.22) between dynamic steady-state balance (i.e., alternating knee raise) and explosive force (i.e., RFD leg extensors) (Table 1). In addition, Figs. 9 and 10 illustrate the associations of proactive balance with maximal strength and muscle power, respectively. Weighted mean *r*_z_ values amounted to 0.47 for measures of maximal strength (*I*^2^ = 47 %, *χ*^2^ = 13.16, *df* = 7, *p* = 0.07, eight studies [53, 54, 57–59, 61–63]) and 0.40 for outcomes of muscle power (*I*^2^ = 0 %, *χ*^2^ = 1.02, *df* = 2, *p* = 0.60, three studies [56, 59, 62]). Back-transformed *r* values of 0.44 and 0.38 indicated small-sized correlations. Furthermore, a small-sized association (*r*_z_ = 0.22, *r* = 0.22) was found between proactive balance (i.e., timed-up-and-go-test) and explosive force (i.e., MVC knee extensors) in one study only [63] (Table 1). Lastly, associations of reactive balance with maximal strength, explosive force, and muscle power are shown in Figs. 5d, 6d, and 7c, respectively. Weighted mean *r*_z_ values amounted to 0.35 for measures of maximal strength (*I*^2^ = 0 %, *χ*^2^ = 1.64, *df* = 2, *p* = 0.44, three studies [37, 59, 65]), 0.49 for variables of explosive force (*I*^2^ = 0 %, *χ*^2^ = 0.14, *df* = 1, *p* = 0.71, two studies [37, 42]), and 0.17 for outcomes of muscle power (*I*^2^ = 0 %, *χ*^2^ = 0.64, *df* = 1, *p* = 0.43, two studies [37, 59]). Back-transformed *r* values of 0.34, 0.45, and 0.17 indicated small-sized correlations.

#### Age Differences

Table 2 shows the comparison of correlation coefficients between children, young adults, and old adults. Statistically significant differences between age groups were obtained for the associations of measures of dynamic steady-state balance with maximal strength only. More precisely, the *r* value in children (*r* = 0.57) was significantly larger than that in young (*r* = 0.09, *z* = 3.30, *p* = 0.001) and old (*r* = 0.35, *z* = 2.94, *p* = 0.002) adults. Further, the *r* value in old adults was significantly larger (*z* = 1.95, *p* = 0.03) than that in young adults.

**Table 2:** Comparison of correlation coefficients between children, young adults, and old adults

Comparison	sSSB	dSSB	RB
Maximal strength			
Children vs. young adults	0.52 (0.30)	3.30 (0.001)	0.38 (0.35)
Children vs. old adults	1.23 (0.13)	2.94 (0.002)	1.05 (0.15)
Young vs. old adults	0.68 (0.25)	1.95 (0.03)	0.54 (0.29)
Explosive force			
Children vs. young adults	NA	NA	NA
Children vs. old adults	NA	NA	NA
Young vs. old adults	NA	NA	0.90 (0.18)
Muscle power			
Children vs. young adults	0.06 (0.48)	NA	0.66 (0.26)
Children vs. old adults	0.33 (0.37)	NA	0.05 (0.48)
Young vs. old adults	0.50 (0.31)	NA	0.51 (0.30)

Data are presented as *z* values with *p* values in parentheses. For proactive balance, no comparison was performed due to an insufficient number of studies available

*dSSB* dynamic steady-state balance, *NA* not available, *RB* reactive balance, *sSSB* static steady-state balance

## Discussion

The present systematic review and meta-analysis characterized and quantified associations between measures of balance and lower-extremity muscle strength/power in healthy individuals across the lifespan. We hypothesized large-sized correlations between proxies of balance and strength/power of the lower extremities, which is based on the premise that similar neurophysiological structures (e.g., activation of corticospinal pathways during perturbed stance and explosive force production) are responsible for the control of balance and lower-extremity muscle strength/power [9–12]. Moreover, transfer effects in terms of training-induced improvements from balance training to an unpracticed strength task, and vice versa, were reported in the literature [10, 13, 14], indicating an interaction between these neuromuscular components. However, our analyses revealed predominately small-sized correlations between variables of balance and muscle strength/power of the lower extremities, which is why our initial hypothesis has to be rejected. This finding was independent from the investigated balance (i.e., static/dynamic steady-state, proactive, reactive balance), strength (i.e., maximal strength, explosive force), and power (e.g., jumps) components.

What are likely explanations for the observed small-sized correlations between measures of balance and lower-extremity muscle strength/power? First, although similar neurophysiological mechanisms (e.g., activation of corticospinal pathways) are involved in the regulation of balance (i.e., perturbed stance) and strength (i.e., explosive force production), it seems that their function during balance control and strength/power production is task specific [9–12]. Indeed, studies investigating spinal and corticospinal excitability during the execution of a strength- or balance-related task showed different activation patterns. For example, short-latency responses induced by transcranial magnetic stimulation were facilitated during the execution of isometric ankle dorsi- and plantarflexions [66] but were unchanged when performing a reactive balance task (i.e., perturbed stance) [67].

Second, transfer effects in terms of strength gains after balance training and balance gains after strength training were reported in the literature [9, 10, 13, 14], yet the underlying adaptations were found to be task specific [9, 10]. For example, Gruber et al. [9, 10] investigated the effects of 4 weeks of balance compared to ballistic strength training on measures of strength using biomechanical (i.e., maximum isometric ankle plantarflexor strength) and electrophysiological (i.e., surface electromyography [EMG], H-reflex, and stretch reflex recording) testing equipment in healthy young adults. The authors reported significant improvements in maximal RFD [9, 10] following both balance and ballistic strength training. However, significant differences in muscle activation and spinal reflex excitability during the execution of strength tasks were found between the two training regimens. More specifically, ballistic strength, but not balance training, resulted in significant increases in EMG activities of the soleus and gastrocnemius muscle during the execution of maximal isometric ankle plantarflexions [9]. In contrast, balance training produced significantly lower peak-to-peak amplitudes of soleus stretch reflexes when performing fast dorsiflexions, whereas no changes occurred after ballistic strength training. In addition, the ratio of the maximum H-reflex to the maximum efferent motor response (*H*_max_:*M*_max_) was significantly reduced following balance training but not after ballistic strength training [10]. Thus, the authors concluded that different neural mechanisms are responsible for similar improvements in measures of lower-extremity muscle strength following balance compared to ballistic strength training.

Third, meta-analyses reflect the highest level on the evidence-based medicine pyramid as compared with original research work [68]. More specifically and in accordance with the preferred reporting items for systematic reviews and meta-analysis (PRISMA) statement guidelines [69], findings from studies that investigated associations between proxies of balance and lower-extremity muscle strength/power and fulfilled predefined selection criteria (e.g., reported a least one measure of balance and strength/power of the lower extremities) were extracted and aggregated. However, a limitation of our meta-analytical approach is that the potential moderating effect of age on associations between the variables of interest (i.e., balance and lower-extremity strength/power) cannot be directly studied. In other words, the influence of age cannot be separated from those of other factors (e.g., sex, training status). Thus, the present findings are preliminary and have to be interpreted with caution. To further our knowledge in this area, studies should be conducted that examine associations between measures of balance and lower-extremity muscle strength/power and that control for potential moderator variables such as age, sex, and training status. This could be realized by conducting a single study that considers, for example, the potential moderating effect of age on the relationship between balance and muscle strength/power in different age groups (i.e., children, adolescents, and young, middle-aged, and old adults).

Furthermore, we hypothesized that age has an impact on the associations between measures of balance and lower-extremity muscle strength/power. As a result, we can partially confirm our second hypothesis. Significant age differences were found for associations between measures of dynamic steady-state balance and maximal strength. More specifically, correlations were larger in children than in young and old adults as well as in old than in young adults. However, the analyses failed to detect further significant age differences in the relationship between other components of balance and strength/power of the lower extremities. Thus, it can be postulated that maturational and biological aging processes of the neuromuscular system may have an influence on the associations between balance and lower-extremity muscle strength/power that is limited to the relationship of dynamic steady-state balance with maximal strength. As a consequence, further research is needed to determine whether age-related differences are specific to the observed associations between measures of dynamic steady-state balance and maximal strength of the lower extremities or if they could also be detected for other proxies of balance and lower-extremity strength/power. To control for this issue, the same test equipment/procedure and outcome measures should be used when comparing different age groups.

A possible reason for the larger correlations between measures of dynamic steady-state balance and maximal strength in children and old adults than in young adults could be caused by differences in the level of task automation. In this regard, previous research [70, 71] showed that during stages of less movement automation (i.e., early in practice or low levels of movement experience), the control of a motor task is relatively unspecific. In other words, muscle selection, computation, and their sequenced activation is not effectively developed and therefore coded on a rather abstract level [72]. As a consequence, the execution of movements with different task characteristics (e.g., balance task vs. strength/power task) can easily be performed, as is shown by larger correlation coefficients. Despite the fact that the performance level of the executed task is low, the result of the movement outcome is more stable because the motor program does not have to be specifically coded. This might be a likely scenario in children and old adults because both age groups show reduced levels of motor control either due to maturation (i.e., children) or biological aging (i.e., old adults) [16–21]. However, with an increasing level of movement automation (i.e., late in practice or high levels of movement experience), the control of a motor task becomes more specific [72]. This is achieved through appropriate movement coding, which results in high performance levels. However, as movement control becomes more specific, the ability to switch between different tasks and their execution is reduced, which is reflected in lower correlation coefficients. Even though performance levels are high, movement execution is more susceptible (less stable) because motor programs have to be coded to achieve relatively specific (more automated) movements. This scenario is likely for young adults because their neuromuscular system is fully developed and enables adequate muscle selection and computation to achieve the desired activation sequence.

Despite the fact that significant age differences were found for the association between measures of dynamic steady-state balance with maximal strength, their size was still small (i.e., *r* values ≤0.69), as was observed for the relationship between other components of balance and lower-extremity muscle strength/power. In general, this indicates that these components are independent of each other (i.e., task specific) and should therefore be tested and trained complementarily across the lifespan. More specifically, testing of individuals at risk of suffering injuries and/or falls should include the assessment of balance and muscle strength/power. For example, Granacher et al. [22] provided recommendations for the assessment of balance and muscle strength/power in healthy older adults. In terms of balance assessment, they recommend that tests for dynamic steady-state (e.g., time/speed while walking 10 m) and reactive (e.g., push-and-release test) balance should be primarily conducted because falls predominantly occur during ambulation and balance perturbations [73]. With respect to the assessment of muscle strength/power, Granacher and colleagues [22] further recommend the application of tests for the assessment of muscle power (e.g., plyometric tests such as CMJ or more functional tests such as the five times chair rise test) because muscle power is more strongly associated with performance in everyday activities (e.g., rising from a chair, stair climbing) as compared with muscle strength [74].

With regards to the implications for training, our findings indicate that programs including all three components (i.e., balance, strength, and power) should be conducted to increase balance and muscular strength/power. This is supported by a study of Lacroix A, Kressig RW, Muehlbauer T, et al. unpublished data who conducted a combined balance and strength/power training program in healthy older adults (age range: 65–80 years). The program included task-specific exercises to improve static/dynamic steady-state, proactive, and reactive balance and strength/power of the lower extremities. Following 12 weeks of training (three times per week), the authors observed significant improvements in measures of static (i.e., Romberg test) and dynamic (i.e., 10-m walk test) steady-state, proactive (i.e., timed-up-and-go test, functional reach test), and reactive balance (e.g., push-and-release test) as well as in lower-extremity muscle strength (i.e., chair rise test) and power (i.e., stair-ascent-and-descent test).

### Limitations

A limitation of the systematic review and meta-analysis is that correlative studies were analyzed representing cross-sectional designs. Therefore, cause and effect relations cannot be deduced. In addition, the investigated associations could be affected by other variables such as joint flexibility, muscle mass, and/or auditory/visual acuity. Further, the observed age-related effects regarding the association between dynamic steady-state balance and maximal strength could be biased due to differences in the number of studies available on that issue for children (two studies), young adults (two studies), and old adults (16 studies). As a consequence, further research is needed to determine whether age influences the relationship between balance and lower-extremity muscle strength/power. For example, an intergenerational approach could be used that incorporates children, adolescents, and young, middle-aged, and old adults when testing components of balance and lower-extremity muscle strength/power. The obtained results should be analyzed using traditional methods (i.e., Pearson product-moment correlation) as well as by applying more sophisticated statistical models such as regression analysis to examine the specific role of age. Moreover, such analyses have to be controlled for potential confounding factors such as the test condition and test parameter.

## Conclusions

The present systematic review and meta-analysis revealed predominately small-sized correlations between measures of balance and lower-extremity muscle strength/power in children, adolescents, and young, middle-aged, and old adults. Significantly different but still small-sized correlation coefficients (i.e., larger *r*-value in children than in young and old adults as well as in old than in young adults) were found for associations between measures of dynamic steady-state balance and maximal strength. Our findings indicate that balance and muscle strength/power of the lower extremities are independent of each other and should therefore be tested (i.e., identification of people at risk of suffering injuries and/or falls) and trained (i.e., development of injury- and fall-prevention programs) complementarily across the lifespan. Further, it appears that maturational processes and biological aging of the neuromuscular system may have an effect on the associations between selected components of balance and lower-extremity muscle strength (e.g., the relationship of dynamic steady-state balance with maximal strength).
